# Machine Learning–Based Early Warning Systems for Clinical Deterioration: Systematic Scoping Review

**DOI:** 10.2196/25187

**Published:** 2021-02-04

**Authors:** Sankavi Muralitharan, Walter Nelson, Shuang Di, Michael McGillion, PJ Devereaux, Neil Grant Barr, Jeremy Petch

**Affiliations:** 1 Centre for Data Science and Digital Health Hamilton Health Sciences Hamilton, ON Canada; 2 DeGroote School of Business McMaster University Hamilton, ON Canada; 3 Dalla Lana School of Public Health University of Toronto Toronto, ON Canada; 4 School of Nursing McMaster University Hamilton, ON Canada; 5 Population Health Research Institute Hamilton, ON Canada; 6 Departments of Health Evidence and Impact and Medicine McMaster University Hamilton, ON Canada; 7 Health Policy and Management DeGroote School of Business McMaster University Hamilton, ON Canada; 8 Institute of Health Policy, Management and Evaluation University of Toronto Toronto, ON Canada; 9 Department of Medicine Faculty of Health Sciences McMaster University Hamilton, ON Canada

**Keywords:** machine learning, early warning systems, clinical deterioration, ambulatory care, acute care, remote patient monitoring, vital signs, sepsis, cardiorespiratory instability, risk prediction

## Abstract

**Background:**

Timely identification of patients at a high risk of clinical deterioration is key to prioritizing care, allocating resources effectively, and preventing adverse outcomes. Vital signs–based, aggregate-weighted early warning systems are commonly used to predict the risk of outcomes related to cardiorespiratory instability and sepsis, which are strong predictors of poor outcomes and mortality. Machine learning models, which can incorporate trends and capture relationships among parameters that aggregate-weighted models cannot, have recently been showing promising results.

**Objective:**

This study aimed to identify, summarize, and evaluate the available research, current state of utility, and challenges with machine learning–based early warning systems using vital signs to predict the risk of physiological deterioration in acutely ill patients, across acute and ambulatory care settings.

**Methods:**

PubMed, CINAHL, Cochrane Library, Web of Science, Embase, and Google Scholar were searched for peer-reviewed, original studies with keywords related to “vital signs,” “clinical deterioration,” and “machine learning.” Included studies used patient vital signs along with demographics and described a machine learning model for predicting an outcome in acute and ambulatory care settings. Data were extracted following PRISMA, TRIPOD, and Cochrane Collaboration guidelines.

**Results:**

We identified 24 peer-reviewed studies from 417 articles for inclusion; 23 studies were retrospective, while 1 was prospective in nature. Care settings included general wards, intensive care units, emergency departments, step-down units, medical assessment units, postanesthetic wards, and home care. Machine learning models including logistic regression, tree-based methods, kernel-based methods, and neural networks were most commonly used to predict the risk of deterioration. The area under the curve for models ranged from 0.57 to 0.97.

**Conclusions:**

In studies that compared performance, reported results suggest that machine learning–based early warning systems can achieve greater accuracy than aggregate-weighted early warning systems but several areas for further research were identified. While these models have the potential to provide clinical decision support, there is a need for standardized outcome measures to allow for rigorous evaluation of performance across models. Further research needs to address the interpretability of model outputs by clinicians, clinical efficacy of these systems through prospective study design, and their potential impact in different clinical settings.

## Introduction

Patient deterioration and adverse outcomes are often preceded by abnormal vital signs [1-3]. These warning signs frequently appear a few hours to a few days before the event, which can provide sufficient time for intervention. In response, clinical decision support early warning systems (EWS) have been developed that employ periodic observations of vital signs along with a predetermined criteria or cut-off range for alerting clinicians of patient deterioration [4].

EWS typically employ heart rate (HR), respiratory rate (RR), blood pressure (BP), peripheral oxygen saturation (SpO_2_), temperature, and sometimes the level of consciousness [5]. Aggregate-weighted EWS incorporate several vital signs and other patient characteristics with clearly defined thresholds. Weights are assigned to each of these vital signs and characteristics based on a threshold, and an overall risk score is calculated by adding each of the weighted scores [6].

Some of the commonly used aggregate-weighted EWS for predicting cardiorespiratory insufficiency and mortality are the Modified Early Warning Score (MEWS) [7], National Early Warning Score (NEWS) [8], and Hamilton Early Warning Score [9], which all incorporate vital signs and the level of consciousness (Alert, Verbal, Pain, Unresponsive [AVPU]) but have varying thresholds for assigning scores.

The predictive ability of aggregate-weighted EWS has limitations. First, the scores indicate the present risk of the patient but do not incorporate trends nor provide information about the possible risk trajectory [6]; thus, the scores do not communicate whether the patient is improving or deteriorating and the rate of this change [10]. Second, these scores do not capture any correlations between the parameters, as the score for each parameter is calculated independently through simple addition [6] (eg, HR or RR can be interpreted differently when body temperature is taken into consideration).

A newer approach to EWS relies on machine learning (ML). ML models learn patterns and relationships directly from data rather than relying on a rule-based system [11]. Unlike aggregate-weighted EWS, ML models are computationally intensive, but can incorporate trends in risk scores, adjust for varying numbers of clinical covariates, and be optimized for different care settings and populations [12]. Like other EWS, ML models can be integrated into electronic health records to analyze vital sign measurements continuously and provide predictions of patient outcomes as part of a clinical decision support system [13].

Two systematic reviews in 2019 [14,15] evaluated the ability of ML models to predict clinical deterioration in adult patients using vital signs. The review by Brekke et al [15] examined the utility of trends within intermittent vital sign measurements from adult patients admitted to all hospital wards and emergency departments (ED) but identified only 2 retrospective studies that met their inclusion criteria. The review identified that vital sign trends were of value in detecting clinical deterioration but concluded that there is a lack of research in intermittently monitored vital sign trends and highlighted the need for controlled trials.

The review conducted by Linnen et al [14] compared the accuracy and workload of ML-based EWS with that of aggregate-weighted EWS. This review focused on studies that reported adult patient transfers to intensive care units (ICUs) or mortality as the outcome(s) and excluded all other clinical settings; 6 studies were identified that reported the performance metrics for both the ML-based EWS and aggregate-weighted EWS. The review identified that ML modelling consistently performed better than aggregate-weighted models while generating clinical workload. They also highlighted the need for standardized performance metrics and deterioration outcome definitions.

These are important findings, but to date no review has systematically reviewed the evidence from studies using ML-based EWS using vital sign measurements of varying frequencies, across different care settings and clinical outcomes in order to identify common methodological trends and limitations with current approaches to generate recommendations for future research in this area.

The objective of this study was to scope the state of research in ML-based EWS using vital signs data for predicting the risk of physiological deterioration in patients across acute and ambulatory care settings and to identify directions for future research in this area.

## Methods

A systematic scoping review was conducted by following the Preferred Reporting Items for Systematic Reviews and Meta-Analyses (PRISMA) extension for scoping reviews (PRISMA-ScR) framework [16]. This process provides an analysis of the available research, current state of utility of ML-based EWS, challenges facing their clinical implementation, and how they compare to aggregate-weighted EWS by identifying, synthesizing, and appraising the relevant evidence in the area. The literature search, assessment of eligibility of full-text articles, inclusion in the review, and extraction of study data were carried out by a single author.

### Search Strategy

We searched PubMed, CINAHL, Cochrane Library, Web of Science, Embase, and Google Scholar for peer-reviewed studies without using any filters for study design and language. Searches were also conducted without any date restrictions. The reference lists of all studies that met the inclusion criteria were screened for additional articles. The search strategy involved a series of searches using a combination of relevant keywords and synonyms, including “vital signs,” “clinical deterioration,” and “machine learning.” See Multimedia Appendix 1 for search terms.

### Eligibility Criteria

The inclusion criteria covered the following:

- Peer-reviewed studies evaluating continuous or intermittent vital sign monitoring in adult patients so that all data collection or sampling frequencies (eg, 1 measurement per minute vs 1 measurement every 2 hours) wedre taken into consideration;
- Studies conducted using data gathered from all acute and ambulatory care settings including medical or surgical hospital wards, ICUs, step-down units, ED, and in-home care;
- Quantitative, observational, retrospective, and prospective cohort studies and randomized controlled trials;
- Studies that involved ML or multivariable statistical or ML models and reported some model performance measure (eg, area under the curve) [17];
- Studies that reported mortality or any outcomes related to clinical deterioration so that EWS models and performance can be examined for all explored outcomes.

The exclusion criteria included the following:

- Studies that used any laboratory values as predictors for the ML-based EWS, as this review focuses on examining time-sensitive predictions of clinical deterioration using patient parameters that are readily available across all care settings;
- Studies involving pediatric or obstetric populations due to these patients having different or altered physiologies that cannot be compared to standard adult patients;
- Qualitative studies, reviews, preprints, case reports, commentaries, or conference proceedings.

### Study Selection

References from the preliminary searches were handled using Mendeley reference management software. After duplicates were removed, titles and abstracts were screened to assess preliminary eligibility. Eligible studies were then read in full length to be assessed against the inclusion and exclusion criteria.

### Data Extraction

Data were extracted from eligible studies using an extraction sheet that followed the PRISMA [18] and Cochrane Collaboration guidelines for systematic reviews [19] and the Transparent Reporting of a Multivariable Prediction Model for Individual Prognosis or Diagnosis (TRIPOD) guidelines [20] for the reporting of predictive models. Study characteristics, setting, demographics, patient outcomes, ML model characteristics, and model performance data were extracted. The model performance results were extracted from the validation data set rather than from the model derivation or training data set to decrease the potential for model overfitting. When studies explored multiple ML models, the model with the best performance was selected for reporting and comparison. If studies compared the performance of ML models to aggregate-weighted EWS, then the performance data of these warning systems were also extracted.

## Results

### Search Results and Study Selection

**Figure 1:**
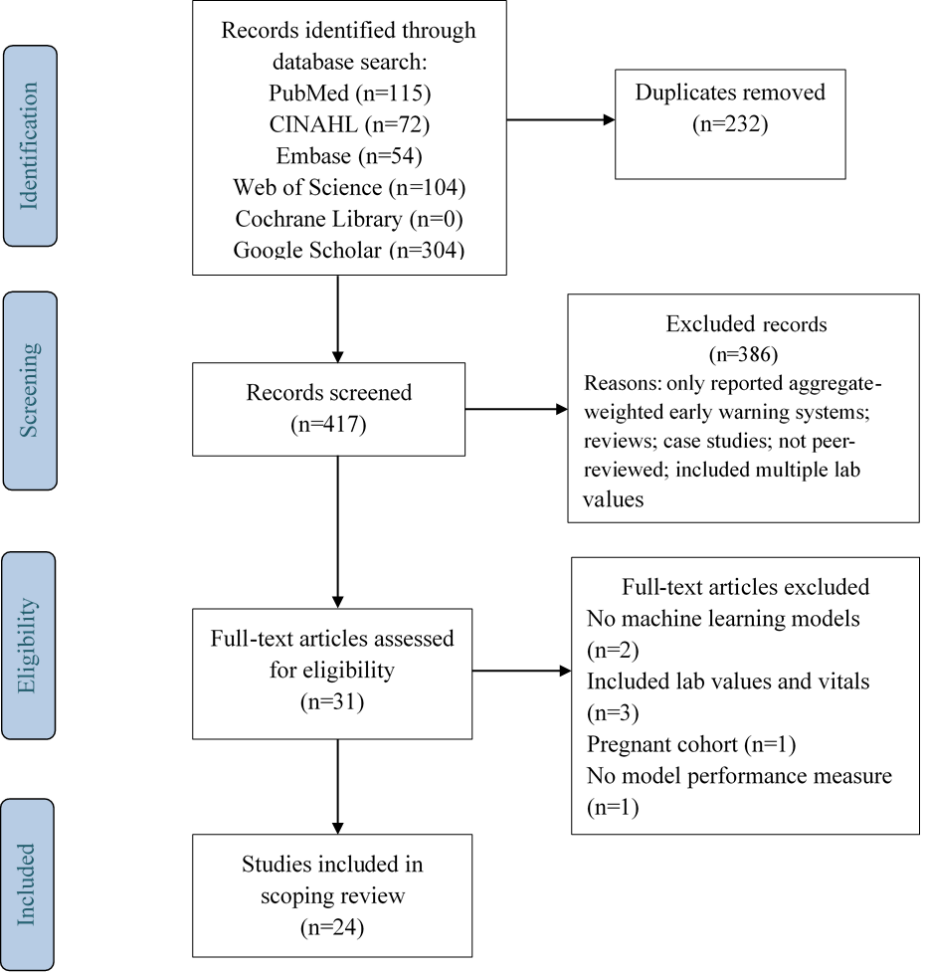
PRISMA flowchart of the search strategy and study selection.

The search for “vital signs” AND “clinical deterioration” AND “machine learning” using the same query terms and filters identified 417 studies after duplicate removal. During the title and abstract screening process, 386 studies were excluded. Of the 31 full-text articles that were assessed, 7 studies were excluded for not meeting the eligibility criteria: 2 studies did not use ML models to predict deterioration, 3 studies included vital sign measurements in addition to laboratory values as predictors, 1 study focused on a cohort of pregnant women, and 1 study did not meet the criteria for model performance measures. A review of the reference lists of the 24 selected studies did not yield any additional studies fulfilling the eligibility criteria (refer to Figure 1).

### Study Characteristics

Of the selected studies, 23 conducted a retrospective analysis of the vital signs data, while 1 study [21] used a prospective cohort study design. Seventeen studies only analyzed continuous vital signs measurements collected through wearable devices and bedside monitors, whereas 3 [22-24] studies analyzed vital signs that were collected both manually and intermittently by clinical staff. Two studies [25,26] analyzed vital signs that were collected both continuously and intermittently, while the remaining 2 studies did not report how the vital sign data were collected.

Studies were conducted in a variety of settings within hospitals while the study by Larburu et al [22] was conducted in an ambulatory setting. While 3 studies [27-29] aimed to develop a remote home-based monitoring tool, the vital sign data used were obtained from the Medical Information Mart for Intensive Care (MIMIC and MIMIC-II) databases [30,31] consisting of data captured from patient monitors in different ICUs. Regarding location, 5 studies [24,26,32-34] were conducted on general wards, 4 studies [11,23,35,36] were conducted in EDs, 7 studies [26,34,37-41] were conducted in ICUs, 2 studies [25,42] were conducted in postoperative wards, and 4 studies [21,43-45] in acute stay wards (medical admission unit, step-down units). Cohort sizes for the studies ranged from 12 patients [39] to 10,967,518 patient visits [11] (refer to Table 1).

**Table 1:** Study characteristics.

Authors, year	Setting(s)	Data collection	Cohort description	Event rate	Study purpose	Predictors	Measurement frequency	Outcome
Badriyah et al, 2014 [45]	Medical assessment unit for 24 hours	Personal digital assistants running VitalPAC software	35,585 admissions	199 (0.56%), cardiac arrest; 1161 (3.26%) unanticipated ICU^a^ admissions; 1789 (5.02%) deaths; 3149 (8.85%) any outcome	Compare the performance of a decision tree analysis with NEWS^b^	HR^c^, RR^d^, SBP^e^, temperature, SpO_2_, AVPU^f^ level, % breathing air at the time of SpO_2_ measurement	Not specified	Cardiac arrest, unanticipated ICU admission, or death, each within 24 hours of a given vital sign observation
Chen at al, 2017 [44]	Step-down unit	Bedside monitors	1880 patients (1971 admissions)	997 patients (53%) or 1056 admissions (53.6%) who experienced CRI^g^ events	Describe the dynamic and personal character of CRI risk evolution observed through continuous vital sign monitoring of individual patients	HR, RR, SPO_2_ (at 1/20 Hz), SBP, DBP^h^	Every 2 hours	CRI
Churpek et al, 2016 [24]	All wards at the University of Chicago and 4 North Shore University Health System hospitals	Data collected manually, documented electronically	269,999 admissions	16,452 outcomes (6.09%)	Whether adding trends improves accuracy of early detection of clinical deterioration and which methods are optimal for modelling trends	Temperature, HR, RR, SpO_2_, DBP, SBP	Every 4 hours	Development of critical illness on the wards: deaths, cardiac arrest, ICU transfers
Chiew et al, 2019 [23]	ED^i^ at Singapore general hospital	Measurements at triage; hospital EHR^j^	214 patients	40 patients (18.7%) met outcome	Compare the performance of HR variability–based machine learning models vs conventional risk stratification tools to predict 30-day mortality	Age, gender, ethnicity, temperature, HR, RR, SBP, DBP, GCS^k^, HR variability	At triage	30-day mortality due to sepsis
Chiu et al, 2019 [42]	Postoperative surgical wards at ﻿4 UK adult cardiac surgical centers	VitalPac ﻿to electronically capture patients’ vital signs	﻿Adults undergoing risk-stratified major cardiac surgery, n=13,631	578 patients (4.2%) with an outcome; 499 ﻿patients (3.66%) with unplanned ICU readmissions	Using logistic regression to model the association of NEWS variables with a serious patient event in the subsequent 24 hours; secondary objectives: comparing the discriminatory power of each model for events in the next 6 hours or 12 hours	RR, SpO_2_, SBP, HR, temperature, consciousness level	Not specified	Death, cardiac arrest, unplanned ICU readmissions
Clifton et al, 2014 [25]	Postoperative ward of the cancer center, ﻿Oxford University Hospitals NHS^l^ Trust, United Kingdom	Continuous vitals monitored by wearable devices; intermittent vitals monitored manually by ward staff	200 patients in the postoperative ward following upper gastrointestinal cancer surgery	Not specified	Using continuous vitals monitoring to provide early warning of physiological deterioration, such that preventative clinical action may be taken	SpO_2_, HR (256 Hz), BP, RR	Continuously (SpO_2_, HR), intermittently (BP, RR)	Physiological deterioration
Desautels et al, 2016 [37]	Beth Israel Deaconess Medical Center ICU	ICU bedside monitors and medical records (MIMIC^m^-III)	22,853 ICU stays	2577 (11.28%) stays with confirmed sepsis	Validate a sepsis prediction method, InSight, for the new Sepsis-3 definitions and make predictions using a minimal set of variables	GCS, HR, RR, SpO_2_, temperature, invasive and noninvasive SBP and DBP	At least 1 measurement per hour	Onset of sepsis
Forkan et al, 2017 [28]	Beth Israel Deaconess Medical Center ICU	ICU bedside monitors and medical records (MIMIC-II)	1023 patients	Not specified	Develop a probabilistic model for predicting the future clinical episodes of a patient using observed vital sign values prior to the clinical event	HR, SBP, DBP, mean BP, RR, SpO_2_	All samples converted to per-minute sampling	Abnormal clinical events
Forkan et al, 2017 [27]	Beth Israel Deaconess Medical Center ICU	ICU bedside monitors and medical records (MIMIC & MIMIC-II)	85 patients	Not specified	Develop an intelligent method for personalized monitoring and clinical decision support through early estimation of patient-specific vital sign values	HR, SBP, DBP, mean BP, RR, SpO_2_	Per-minute sampling	Patient-specific anomalies, disease symptoms, and emergencies
Forkan et al, 2017 [29]	Beth Israel Deaconess Medical Center ICU	ICU bedside monitors and medical records (MIMIC-II)	4893 patients	Not specified	Build a prognostic model, ViSiBiD, that can accurately identify dangerous clinical events of a home-monitored patient in advance	HR, SBP, DBP, mean BP, RR, SpO_2_	Per-minute sampling	Dangerous clinical events
Guillame-Bert et al, 2017 [43]	Step-down unit	Bedside monitor measurements over 8 weeks	297 admissions	127 patients (43%) ﻿exhibited at least 1 real event during their stay	Forecast CRI utilizing data from continuous monitoring of physiologic vital sign measurements	HR, RR, SPO_2_, SBP, DBP, mean BP	Every 20 seconds (HR, RR, SPO_2_), every 2 hours (SBP, DBP, and mean BP)	At least 1 event threshold limit criteria exceeded for >80% of last 3 minutes
Ho et al, 2017 [38]	Beth Israel Deaconess Medical Center ICU	ICU bedside monitors and medical records (MIMIC-II)	763 patients	197 patients (25.8%) experienced a cardiac arrest event	Build a cardiac arrest risk prediction model capable of early notification at time z (z ≥5 hours prior to the event)	Temperature, SpO_2_, HR, RR, DBP, SBP, pulse pressure index	1 reading per hour	Cardiac arrest
Jang et al, 2019 [35]	ED visits to a tertiary academic hospital	EHR data from ED visits	Nontraumatic ED visits	374,605 eligible ED visits of 233,763 patients; 1097 (0.3%) patients with cardiac arrest	Develop and test artificial neural network classifiers for early detection of patients at risk of cardiac arrest in EDs	Age, sex, chief complaint, SBP, DBP, HR, RR, temperature, AVPU	Not specified	Development of cardiac arrest within 24 hours after prediction
Kwon et al, 2018 [26]	Cardiovascular teaching hospital and community general hospital	Data collected manually by staff on general wards, by bedside monitors in ICUs	52,131 patients	419 patients (0.8%) with cardiac arrest; 814 (1.56%) deaths without attempted resuscitation	Predict whether an input vector belonged within the prediction time window (0.5-24 hours before the outcome)	SBP, HR, RR, temperature	3 times a day on general wards, every 10 minutes in ICUs	Primary outcome: first cardiac arrest; secondary outcome: death without attempted resuscitation
Kwon et al, 2018 [11]	﻿151 EDs in Korea	Korean National Emergency Department Information System (NEDIS)	﻿10,967,518 ED visits﻿	153,217 (1.4%) in-hospital deaths; 625,117 (5.7%) critical care admissions; 2,964,367 (27.0%) hospitalizations	Validate that a DTAS^n^ identifies high-risk patients more accurately than existing triage and acuity scores	Age, sex, chief complaint, time from symptom onset to ED visit, arrival mode, trauma, initial vital signs (SBP, DBP, HR, RR, temperature), mental status	At ED admission	﻿Primary outcome: in-hospital mortality; secondary outcome: critical care; tertiary outcome: hospitalization
Larburu et al, 2018 [22]	﻿OSI Bilbao-Basurto (Osakidetza) Hospital and ED admissions, ambulatory	Collected manually by clinicians and patients	242 patients	202 predictable decompensations	Prevent mobile heart failure patients’ decompensation using predictive models	SBP, DBP, HR, SaO_2_, weight	At diagnosis and 3-7 times per week in ambulatory patients	Heart failure decompensation
Li et al, 2016 [39]	Beth Israel Deaconess Medical Center ICU	ICU bedside monitors and medical records (MIMIC-II)	12 patients	Not specified	Adaptive online monitoring of patients in ICUs	HR, SBP, DBP, MAP^o^, RR	At least 1 measurement per hour	Signs of deterioration
Liu et al, 2014 [36]	﻿ED of a tertiary hospital in Singapore	Manual vital measurements by nurses or physicians	702 patients with undifferentiated, nontraumatic chest pain	29 (4.13%) patients met primary outcome	﻿Discover the most relevant variables for risk prediction of major adverse cardiac events using clinical signs and HR variability	SBP, RR, HR	Not specified	﻿Composite of events such as death and cardiac arrest within 72 hours of arrival at the ED
Mao et al, 2018 [34]	ICU, inpatient wards, outpatient visits	UCSF^p^ dataset:inpatient and outpatient visits; MIMIC-III: ICU bedside monitors	UCSF: 90,353 patients; MIMIC-III: 21,604 patients	UCSF: 1179 (1.3%) sepsis, ﻿349 ﻿(0.39%) severe sepsis, ﻿614 ﻿(0.68%) septic shock; MIMIC-III: ﻿sepsis (1.91%), severe sepsis (2.82%), septic shock (4.36%)	Sepsis prediction	SBP, DBP, HR, RR, SpO_2_, temperature	Hourly	Sepsis, severe sepsis, septic shock
Olsen et al, 2018 [46]	PACU^q^, Rigshospitalet, University of Copenhagen, Denmark	IntelliVue MP5, BMEYE Nexfin bedside monitors during admission to post anesthetic care unit	178 patients	160 (89.9%) had ≥1 microevent occurring during admission; 116 patients (65.2%) had ≥1 microevent with a duration >15 minutes	Develop a predictive algorithm detecting early signs of deterioration in the PACU using continuously collected cardiopulmonary vital signs	SpO_2_, SBP, HR, MAP	Every minute (SpO_2_, SBP, HR), every 15 minutes (MAP)	Signs of deterioration
Shashikumar et al, 2017 [40]	Adult ICU units	ICU bedside monitors, Bedmaster system; up to 24 hours of monitoring	Patients with unselected mixed surgical procedures	242 sepsis cases	Predict onset of sepsis 4 hours ahead of time, using commonly measured vital signs	MAP, HR, SpO_2_, SBP, DBP, RR, GCS, temperature, comorbidity, clinical context, admission unit, surgical specialty, wound type, age, gender, weight, race	≥1 measurement per hour	Onset of sepsis
Tarassenko et al, 2006 [32]	General wards at John Radcliffe Hospital in Oxford, United Kingdom	Bedside monitors for at least 24 hours per patient	150 general-ward patients	Not specified	A real-time automated system, BioSign, which tracks patient status by combining information from vital signs	HR, RR, SpO_2_, skin temperature, average SBP -average DBP	Every 30 minutes (BP), every 5 seconds (other vitals)	Signs of deterioration
Van Wyk et al, 2017 [33]	Methodist LeBonheur Hospital, Memphis, TN	Bedside monitors: Cerner CareAware iBus system	2995 patients	343 patients (11.5%) diagnosed with sepsis	Classify patients into sepsis and nonsepsis groups using data collected at various frequencies from the first 12 hours after admission	HR, MAP, DBP, SBP, SpO_2_, age, race, gender, fraction of inspired oxygen	Every minute	Sepsis detection
Yoon et al, 2019 [41]	Beth Israel Deaconess Medical Center ICU	ICU bedside monitors and medical records (MIMIC-II)	2809 subjects	787 tachycardia episodes	Predicting tachycardia as a surrogate for instability	Arterial DBP, arterial SBP, HR, RR, SpO_2_, MAP	1/60 Hz or 1 Hz	Tachycardia episode

^a^ICU: intensive care unit.

^b^NEWS: National Early Warning Score.

^c^HR: heart rate.

^d^RR: respiratory rate.

^e^SBP: systolic blood pressure.

^f^AVPU: alert, verbal, pain, unresponsive.

^g^CRI: cardiorespiratory instability.

^h^DBP: diastolic blood pressure.

^i^ED: emergency department.

^j^EHR: electronic health record.

^k^GCS: Glasgow Coma Score.

^l^NHS: National Health Service.

^m^MIMIC: Medical Information Mart for Intensive Care.

^n^DTAS: Deep learning–based Triage and Acuity Score.

^o^MAP: mean arterial pressure.

^p^UCSF: University of California, San Francisco.

^q^PACU: postanesthesia care unit.

### Predictor Variables

The most commonly used vital sign predictors were HR, RR, systolic BP, diastolic BP, SpO_2_, body temperature, level of consciousness through either the Glasgow Coma Score or the AVPU scale, and mean arterial pressure. Measurement frequencies for these variables ranged from once every 5 seconds [32] in hospital wards to 3-7 times per week [22] in an ambulatory setting. Other commonly used predictors included age, gender, weight, ethnicity, chief complaint, and comorbidities.

### Outcomes

The outcomes being predicted in most studies focused on cardiorespiratory insufficiency–related events. Cardiac arrest was the primary outcome in 7 [24,26,35,36,38,42,45] studies, while general cardiorespiratory deterioration or decompensation was the primary outcome in 5 studies [25,39,41,43,44]. Another commonly predicted outcome was sepsis, which included the time of onset of sepsis [34,37,40], severe sepsis [33,34], septic shock [34], and sepsis-related mortality [23]. Other outcomes explored within the studies include unanticipated ICU admissions [24,42,45], development of critical illness [24], general physiological deterioration [25,32,39,46], abnormal or dangerous clinical events [27-29], and mortality [11,24,42].

Outcomes were first identified, and baseline models were created using predefined parameter thresholds (ground truth) consistent with the MEWS [23,26,35] or NEWS [23,42,46] criteria for cardiorespiratory instability and general physiological deterioration, while the sepsis-related outcomes were identified based on the thresholds set within the systemic inflammatory response syndrome [34], quick Sequential Organ Failure Assessment (qSOFA) [23], and SOFA [37] criteria. Some studies [22,27-29,43,44] also used thresholds and criteria based on the population served by their individual care setting.

### ML Models and Performance

All included studies consider the prediction of deterioration risk to be a classification task and therefore use different types of classification models in the process, including tree-based models, linear models, kernel-based methods, and neural networks (refer to Table 2 for a full inventory of methods used, model performance achieved, and prediction windows, and see Multimedia Appendix 2 for a description of ML methods).

Measures used to assess model performance varied across the studies. The most common measure was the area under the receiver operator characteristic (AUROC) along with model accuracy, sensitivity, and specificity. Area under the precision-recall, F-score, Hamming’s score, and precision (positive predictive value) were reported less commonly.

Prediction windows ranged from 30 minutes to 30 days before an event.

Model performance varied substantially based on outcome measure being predicted (eg, cardiorespiratory insufficiency vs sepsis), ML method used (eg, linear vs tree-based), and prediction window (eg, 30 minutes before an event vs 4 hours before).

**Table 2:** Machine learning (ML) models and comparisons used for outcome prediction.

Study	Cohort	Event rate	ML model(s)	Missing data handling	Best ML model performance	ML model comparisons	Prediction window	Aggregate weighted EWS^a^ comparisons
Badriyah et al, 2014 [45]	35,585 admissions	199 (0.56%), cardiac arrest; 1161 (3.26%) unanticipated ICU^b^ admissions; 1789 (5.02%) deaths; 3149 (8.85%) any outcome	Decision tree analysis	Not specified	Decision tree predicted cardiac arrest: AUROC^c^=0.708; unanticipated ICU admission: AUROC=0.862; death: AUROC=0.899; any outcomes: AUROC=0.877	Not specified	Within 24 hours preceding events	NEWS^d^ AUROC: cardiac arrest, ﻿0.722; unanticipated ICU admission, ﻿0.857; death, ﻿0.894; any outcomes, ﻿0.873
Chen at al, 2017 [44]	1880 patients (1971 admissions)	997 patients (53%) or 1056 admissions (53.6%) who experienced CRI^e^ events	Variant of the random forest classification model using nonrandom splits	Not specified	Random forest AUC^f^ initially remained constant (0.58-0.60), followed by an increasing trend, with AUCs rising from 0.57 to 0.89 during the 4 hours immediately preceding events	Logistic regression: AUC=0.7; lasso logistic regression: AUC=0.82	Within 4 hours preceding events	No comparison
Churpek et al, 2016 [24]	269,999 admissions	16,452 outcomes (6.09%)	Univariate analysis, bivariate analysis	Forward imputation, median value imputation	Trends increased model accuracy compared to a model containing only current vital signs (AUC 0.78 vs 0.74); vital sign slope improved AUC by 0.013	Not specified	Within 4 hours preceding events	No comparison
Chiew et al, 2019 [23]	214 patients	40 patients (18.7%) met outcome	K-nearest neighbor, random forest, adaptive boosting, gradient boosting, support vector machine	Not specified	Gradient boosting predicted 30-day sepsis-related mortality: F1 score=0.50, AUPRC=0.35, precision (PPV^g^)=0.62, recall=0.5	K-nearest neighbor: F1 score=0.10, AUPRC=0.10, precision (PPV)=0.33, recall=0.6; random forest: F1 score=0.35, AUPRC=0.27, precision (PPV)=0.26, recall=0.56; adaptive boosting: F1 score=0.40, AUPRC=0.31, precision (PPV)=0.43, recall=0.38; SVM^h^: F1 score=0.43, AUPRC=0.29, precision (PPV)=0.33, recall=0.63	Within 30 days preceding event	﻿SEDS^i^: F1=0.40, AUPRC=0.22; qSOFA^j^: F1=0.32, AUPRC=0.21; NEWS; F1=0.38, AUPRC=0.28; MEWS^k^: F1=0.30, AUPRC=0.25
Chiu et al, 2019 [42]	Adults undergoing risk-stratified major cardiac surgery (n=13,631)	578 patients (4.2%) with an outcome; 499 ﻿patients (3.66%) with unplanned ICU readmissions	Logistic regression	Observations with missing values were excluded	Logistic regression predicted the event 24 hours in advance: AUROC=0.779; 12 hours in advance: AUROC=0.815; 6 hours in advance: AUROC=0.841	Not specified	Within 24, 12, and 6 hours preceding event	NEWS: 24 hours before event, AUROC=0.754; 12 hours before event, AUROC=0.789; 6 hours before event, AUROC=0.813
Clifton et al, 2014 [25]	200 patients in the postoperative ward following upper gastrointestinal cancer surgery	Not specified	Classifiers, Gaussian process, one-class support vector machine, kernel estimate	Missing channels replaced by mean of that channel	SVM predicted deterioration: accuracy=0.94, partial AUC=0.28, sensitivity=0.96, specificity=0.93	Conventional SVM: accuracy=0.90, partial AUC=0.26, sensitivity=0.92, specificity=0.87; Gaussian mixture models: accuracy=0.9, partial AUC=0.24, sensitivity=0.97, specificity=0.84; Gaussian processes: accuracy=0.90, partial AUC=0.26, sensitivity=0.91, specificity=0.89; kernel density estimate: accuracy=0.91, partial AUC=0.26, sensitivity=0.94, specificity=0.87	Not specified	No comparison
Desautels et al, 2016 [37]	22,853 ICU stays	2577 (11.28%) stays with confirmed sepsis	Insight classifier	Carry forward imputation	Classifier predicts sepsis at onset: AUROC=0.880, APR^l^=0.6, accuracy=0.8; classifier predicts sepsis 4 hours before onset: AUROC=0.74, APR=0.28, accuracy=0.57	Not specified	Within 4 hours preceding event and at time of event onset	SIRS^m^: AUROC= 0.609, APR= 0.160; qSOFA: AUROC= 0.772, APR=0.277; MEWS: AUROC=0.803, APR=0.327; SAPS^n^ II: AUROC=0.700, APR=0.225; SOFA: AUROC=0.725, APR=0.284
Forkan et al, 2017 [28]	1023 patients	Not specified	PCA^o^ used to separate patients into multiple categories; hidden Markov Model adopted for probabilistic classification and future prediction	Data with consecutive missing values over a long period are eliminated	Hidden Markov Model event prediction: accuracy=97.8%, precision=92.3, sensitivity=97.7, specificity=98, F-score=95%	Neural network: accuracy=93%	Within 30 minutes preceding event	No comparison
Forkan et al, 2017 [27]	85 patients	Not specified	Multilabel classification algorithms are applied in classifier design; result analysis with ﻿J48 decision tree, random tree and sequential minimal optimization (SMO, a simplified version of SVM)	Where ≥1 vital signs data are missing while clean values of others are available, considered as recoverable and imputed using median-pass and k-nearest neighbor filter	Predictions across 24 classifier combinations yielded a Hamming score of 90%-95%; F1-micro average of 70.1%-84%; accuracy of 60.5%-77.7%	Not specified	Within 1 hour preceding event	No comparison
Forkan et al, 2017 [29]	4893 patients	Not specified	J48 decision tree, random forest, sequential minimal optimization, MapReduce random forest	Data with consecutive missing values over a long period are eliminated	Event prediction by random forest: within a 60-minute forecast horizon, F score=0.96, accuracy=95.86; within a 90-minute forecast horizon, F-score=0.95, accuracy=95.35; within a 120-minute forecast horizon, F-score=0.95, accuracy=95.18	J48 decision tree: within a 60-minute forecast horizon, F score=0.93, accuracy=92.46; within a 90-minute forecast horizon, F score=0.92, accuracy=91.59; within a 120-minute forecast horizon, F score=0.91, accuracy=91.30; Event prediction with sequential minimal optimization: within a 60-minute forecast horizon, F score=0.91, accuracy=90.72; within a 90-minute forecast horizon, F score=0.90, accuracy=90.08; within a 120-minute forecast horizon, F score=0.89, accuracy=89.23	1 hour preceding event	No comparison
Guillame-Bert et al, 2017 [43]	297 admissions	127 patients (43%) ﻿exhibited at least 1 real CRI event during their stay in the step-down unit	TITA^p^ rules, rule fusion algorithm; mapping function from rule-based features to forecast model learned using random forest classifier	Not specified	Event forecast alert within 17 minutes, 51 seconds before onset of CRI (false alert every 12 hours); event forecast alert within 10 minutes, 58 seconds before onset of CRI (false alert every 24 hours)	Random forest: event forecast alert within 11 minutes, 25 seconds before onset of CRI (false alert every 12 hours); event forecast alert within 5 minutes, 52 seconds before onset of CRI (false alert every 24 hours)	Within 17 minutes, 51 seconds preceding CRI onset	No comparison
Ho et al, 2017 [38]	763 patients	197 patients (25.8%) experienced a cardiac arrest event	Temporal transfer learning-based model (TTL-Reg)	Imputed values based on the median from patients of the same gender and similar ages	TTL-Reg predicts events with an AUC of 0.63	Not specified	Within 6 hours preceding event	No comparison
Jang et al, 2019 [35]	Non-traumatic ED visits	374,605 eligible ED visits of 233,763 patients; 1097 (0.3%) patients with cardiac arrest	ANN^q^ with multilayer perceptron, ANN with LSTM^r^, hybrid ANN; comparison with random forest and logistic regression	Not specified	Event prediction: ANN with multilayer perceptron, AUROC=0.929; ANN with LSTM, AUROC=0.933; hybrid ANN, AUROC=0.936	Random forest, AUROC=0.923; logistic regression, AUROC=0.914	Within 24 hours preceding event	MEWS: AUROC=0.886
Kwon et al, 2018 [26]	52,131 patients	419 patients (0.8%) with cardiac arrest; 814 (1.56%) deaths without attempted resuscitation	3 RNN^s^ layers with LSTM to deal with time series data; compared to random forest and logistic regression	Most recent value was used; if no value available, then median value used	Event prediction: RNNs, AUROC=0.85, AUPRC^t^=0.044	Random forest, AUROC=0.78, AUPRC=0.014; logistic regression, AUROC=0.613, AUPRC=0.007	30 minutes to 24 hours preceding event	MEWS: AUROC=0.603, AUPRC=0.003
Kwon et al, 2018 [11]	﻿10,967,518 ED visits	153,217 (1.4%) in-hospital deaths; 625,117 (5.7%) critical care admissions; 2,964,367 (27.0%) hospitalizations	DTAS^u^ using multilayer perceptron ﻿with 5 hidden layers	Excluded	Event prediction: DTAS using multilayer perceptron, AUROC=0.935, AUPRC=0.264	﻿Random forest: AUROC= 0.89, AUPRC= 0.14; logistic regression: AUROC= 0.89, AUPRC=0.16	Not specified	﻿Korean triage and acuity score: AUROC =0.785, AUPRC=0.192; MEWS: AUROC=0.810, AUPRC=0.116;
Larburu et al, 2018 [22]	242 patients	202 predictable decompensations	Naïve Bayes, decision tree, random forest, SVM	Not specified	Decompensation event prediction: naïve Bayes, AUC=67%	Decision tree, neural network, random forest, support vector machine, stochastic gradient descent	Not specified	No comparison
Li et al, 2016 [39]	12 patients	Not specified	L-PCA (combination of just-in-time learning and PCA)	Not specified	Fault detection rate with L-PCA: 20% higher than with PCA; 47% higher than with fast moving-window PCA; best detection rate achieved was 99.8%	Not specified	Not specified	No comparison
Liu et al, 2014 [36]	702 patients with undifferentiated, non-traumatic chest pain	29 (4.13%) patients met primary outcome	﻿Novel variable selection framework based on ensemble learning; random forest was the independent variable selector for creating the decision ensemble	Not specified	Event prediction with ensemble learning model: AUC=0.812, cut-off score=43, sensitivity=82.8%, specificity=63.4%	Not specified	Within 72 hours of arrival at ED	TIMI^v^: AUC=0.637; MEWS: AUC=0.622
Mao et al, 2018 [34]	UCSF^w^: 90,353 patients; MIMIC^x^-III: 21,604 patients	UCSF: 1179 (1.3%) sepsis, ﻿349 ﻿(0.39%) severe sepsis, ﻿614 ﻿(0.68%) septic shock; MIMIC-III: ﻿sepsis (1.91%), severe sepsis (2.82%), septic shock (4.36%)	Gradient tree boosting + transfer learning using MIMIC-III as source and UCSF as target	Carry forward imputation	Detection with gradient tree boosting: AUROC=0.92 for sepsis; AUROC=0.87 for severe sepsis at onset; AUROC=0.96 for septic shock 4 hours before; AUROC=0.85 for severe sepsis prediction 4 hours before	Not specified	At onset of sepsis and severe sepsis; within 4 hours preceding septic shock and severe sepsis	MEWS: AUROC=0.76; SOFA: AUROC=0.65; SIRS: AUROC=0.72
Olsen et al, 2018 [46]	178 patients	160 (89.9%) had ≥1 microevent occurring during admission; 116 patients (65.2%) had ≥1 microevent with a duration >15 minutes	Random forest classifier	Not specified	Detection of early signs of deterioration with random forest: accuracy=92.2%, sensitivity=90.6%, specificity=93.0%, AUROC=96.9%	Not specified	Not specified	Compared with hospital's current alarm system: number of false alarms decreased by 85%, number of missed early signs of deterioration decreased by 73%
Shashikumar et al, 2017 [40]	Patients with unselected mixed surgical procedures	242 sepsis cases	Elastic net logistic classifier	Median values (if multiple measurement were available); otherwise, the old values were kept (sample-and-hold interpolation); mean imputation for replacing all remaining missing values	Event prediction: elastic net logistic classifier using entropy features alone, AUROC=0.67, accuracy=47%; elastic net logistic classifier using social demographics + EMR^y^ features, AUROC=0.7, accuracy=50%; elastic net logistic classifier using all features, AUROC=0.78, accuracy=61%	Not specified	4 hours prior to onset	No comparison
Tarassenko et al, 2006 [32]	150 general-ward patients	Not specified	﻿Biosign; data fusion method: probabilistic model of normality in five dimensions	Historic, median filtering	﻿95% of Biosign alerts were classified as “True” by clinical experts	Not specified	Within 120 minutes of event	No comparison
Van Wyk et al, 2017 [33]	2995 patients	343 patients (11.5%) diagnosed with sepsis	CNN^z^ (constructed images using raw patient data) with random dropout to reduce overfitting; multilayer perceptron with random dropout between layers to avoid overfitting	Not specified	Event classification with a 1-minute observation frequency: CNN, accuracy=86.1%; event classification with a 10-minute observation frequency: CNN, accuracy=78.2%	Event classification with a 1-minute observation frequency: multilayer perceptron, accuracy=76.5%; event classification with a 10-minute observation frequency: multilayer perceptron, accuracy=71%	Not specified	No comparison
Yoon et al, 2019 [41]	2809 subjects	787 tachycardia episodes	Regularized logistic regression and random forest classifiers	Discrete Fourier transform, cubic-spline interpolation of heart rate and respiratory rate data for missing data as long as ≥20% of the data were available	Event prediction: random forest, AUC=0.869, accuracy=0.806	Logistic regression with L1 regularization, AUC=0.8284, accuracy=0.7668	Within 3 hours preceding onset	No comparison

^a^EWS: early warning system.

^b^ICU: intensive care unit.

^c^AUROC: area under the receiver operator characteristic.

^d^NEWS: National Early Warning Score.

^e^CRI: cardiorespiratory instability.

^f^AUC: area under the curve.

^g^PPV: positive predictive value.

^h^SVM: support vector machine.

^i^SEDS: Singapore Emergency Department Sepsis.

^j^qSOFA: quick Sequential Organ Failure Assessment.

^k^MEWS: Modified Early Warning Score.

^l^APR: area under the precision-recall curve.

^m^SIRS: systemic inflammatory response syndrome.

^n^SAPS II: simplified acute physiology score.

^o^PCA: principal component analysis.

^p^TITA: temporal interval tree association.

^q^ANN: artificial neural network.

^r^LSTM: long short-term memory.

^s^RNN: recurrent neural network.

^t^AUPRC: area under the precision-recall curve.

^u^DTAS: Deep learning–based Triage and Acuity Score.

^v^TIMI: Thrombolysis in Myocardial Infarction.

^w^UCSF: University of California, San Francisco.

^x^MIMIC: Medical Information Mart for Intensive Care.

^y^EMR: electronic medical record.

^z^CNN: convolutional neural network.

### Comparison With Aggregate-Weighted EWS

Nine studies compared the performance of ML-based EWS with aggregate-weighted EWS. Studies exploring cardiorespiratory outcomes, general physiological deterioration, or mortality carried out comparisons with NEWS [42,45], MEWS [11,26,35,36], and the Thrombolysis in Myocardial Infarction score [36]. The 3 studies exploring sepsis-related outcomes additionally included the SOFA, qSOFA, and SIRS criteria and the simplified acute physiology (II) score [23,34,37]. A few studies also drew comparisons with other customized scoring systems individual to their care setting or region such as the Korean Triage and Acuity Score [11], Singapore Emergency Department Sepsis model [23], and postanesthesia care unit alarm system [46].

In all 9 studies, the ML models performed better than the aggregate-weighted EWS systems for all clinical outcomes except for cardiac arrest in the study by Badriyah et al [45]. For example, in the study by Jang et al [35], a long short-term memory neural network achieved an AUROC of 0.933, an improvement over MEWS, which achieved an AUROC of 0.886 using the same data. Similarly, in the study by Kwon et al [26], recurrent neural networks achieved an AUROC of 0.85 compared to 0.603 for MEWS and 0.785 for the Korean Triage and Acuity Score. Some studies reported much more modest improvements, such as the study by Chiu et al [42] that achieved an AUROC of 0.779 using logistic regression, compared to 0.754 using MEWS for the same 24-hour prediction window. A full side-by-side comparison of ML vs aggregate-weighted EWS is presented in Multimedia Appendix 3.

## Discussion

Based on this scoping review, ML-based EWS models show considerable promise, but there exist several important avenues for future research if these models are to be effectively implemented in clinical practice.

### Prediction Window

A model’s prediction window refers to how far in advance a model is predicting an adverse event. Most studies included in our review used a prediction window between 30 minutes [26] and 72 hours [36] before the clinical deterioration took place. The length of a model’s prediction window is important because a prediction window that is too short will not yield any real clinical benefit (it would not give a clinical team sufficient time to intervene), but a number of studies [29,34,37,42] showed a decrease in model performance when the prediction window was longer (eg, AUROC drops from 0.88 at the time of onset to 0.74 at 4 hours before the event). Future research seeking to maximize the clinical benefit of ML EWS should strive to achieve an optimum balance between a clinically relevant prediction window and clinically acceptable model performance, rather than simply maximizing a model performance metric, such as AUROC.

### Clinically Actionable Explanations

The studies included in this review focused on ML model development and did not explore how the output of these models would be communicated to clinicians. Since many ML models are “black boxes” [46,47], it may not be immediately clear to clinicians what the likely reason for an alert might be until the patient is assessed, which can cause further delays in time-sensitive scenarios. However, in the broader ML field, there has been significant recent progress in explainable ML techniques, and it has been pointed out that these approaches may be preferred by the medical community and regulators [48,49]. Several explanation methods take specific, previously black-box methods, such as convolutional neural networks [50], and allow for post-hoc explanation of their decision-making process. Other explainability algorithms are model-agnostic, meaning they can be applied to any type of model, regardless of its mathematical basis [51]. In the study by Lauritsen et al [52], an explainable EWS was developed based on a temporal convolutional network, using a separate module for explanations. These methodologies are promising, but their application to health care, including to EWS, has been limited. Objective evaluation of the utility of explanation methods is a difficult, ongoing problem, but is an important direction for future research in the area of ML-based EWS if they are to be effectively deployed in clinical practice [53].

### Expanded Study Settings

Nearly all the studies included in this review were conducted in inpatient settings. While EWS are highly valuable in an inpatient context, there is also considerable need in the ambulatory setting, particularly postdischarge. For example, the VISION study [54] found that 1.8% of all patients die within 30 days postsurgery and 29.4% of all deaths occurred after patients were discharged from hospital. Patients often receive postoperative monitoring only 3-4 weeks [54] after discharge during a follow-up visit with their surgeon. During this period, it has been shown that many patients suffer from prolonged unidentified hypoxemia [55] and hypotension [56], which are precursors to serious postoperative complications. While EWS research has historically focused on inpatient settings due to the availability of continuous vital signs data, the increasing availability of remote patient monitoring and wearable technologies offer the opportunity to direct future EWS research to the ambulatory setting to address a significant clinical need.

### Retrospective Versus Prospective Evaluation

All but one study [21] included in this review were retrospective in nature, leaving open the possibility that algorithm performance in a clinical environment may be lower than the performance achieved in a controlled retrospective setting [34]. It is also unclear how often these EWS were able to identify clinical deterioration that had not already been detected by a care team. Further, alerts for clinical deterioration may be easily disregarded by clinicians due to alert fatigue, even when the risk of deterioration has been correctly identified [43]. In the single case where an ML-based EWS was studied prospectively, Olsen et al [21] found that the random forest classifier decreased false alarm rates by 85% and the rate of missed alerts by 73% when compared to the existing aggregate-weighted alarm system. While the predictions were independently scored for severity by 2 clinician experts, the interpretation of the clinical impact of these alerts was not explored any further, leaving the question of clinical benefit unanswered. Future research into ML-based EWS should begin to include prospective evaluation, both of model accuracy (to understand how model performance is affected when faced with real-world data) and of clinical outcomes (to understand whether alerts in fact produce clinical benefits).

### Standardizations of Performance Metrics

A key observation from this review is the lack of an agreed-upon standard among the research community for reporting performance measures across studies. This makes meaningful comparison between the outcomes of these studies difficult, and where there is overlap, it is not clear that the most clinically relevant metrics have been chosen. The majority of the studies in this review report the AUROC as the main performance metric, reflecting a common practice in the ML literature. However, AUROC may not be adequate for evaluating the performance of the EWS in a clinical setting [57].

As Romero-Brufau et al [58] discussed in their article, AUROC does not incorporate information about the prevalence of physiological deterioration, which can be lower than 0.02 daily in a general inpatient setting. This can make AUROC a misleading metric, leading to overestimation of clinical benefit and underestimation of clinical workload and resources. [58] When the prevalence is low (<0.1), even a model with high sensitivity and specificity may not yield a high posttest probability for a positive prediction [15]. Therefore, reporting metrics that incorporate the prevalence would be more appropriate.

The performance of an EWS depends on the tradeoff between 2 goals: early detection of outcomes versus issuance of fewer false-positive alerts to prevent alarm fatigue [43]. Sensitivity can be a good metric to evaluate the first goal as it would provide the percentage of true-positive predictions within a certain time period. To evaluate the clinical burden of false-positive alerts, the positive predictive value, which incorporates prevalence, can be used as it gives a percentage of useful alerts that lead to a clinical outcome. The number needed to evaluate can be a useful measure of clinical utility and cost-efficiency of each alert as it provides the number of patients that need to be evaluated further to detect one outcome. Using these metrics to evaluate tradeoffs between outcome detection and workload would be essential for determining the clinical utility of the EWS [58]. Additionally, the F1 score can also be a useful metric as it provides a measure of the model’s overall accuracy through the calculation of the harmonic mean of the precision and recall (sensitivity). Balancing the use of these 2 metrics could yield a more realistic measure of the model’s performance [58].

### Comparison to “Gold Standard” EWS

On a related note, only 9 of the studies included in our review made comparisons between their ML-based models and a “gold standard” aggregate-weighted EWS, such as MEWS or NEWS. Future research in the area should report a commonly used aggregate-weighted EWS as a baseline model, which would aid in making effective comparisons between them. NEWS may be particularly well suited to this area of research as its input variables can all be measured automatically and continuously via devices.

### Strengths of the Review

The search strategy was comprehensive while not being too focused on specific clinical outcomes, sampling frequencies, or filtering for time. This allowed for the identification of as many studies as possible that examined the use of ML models and vital signs to predict the risk of patient deterioration. No additional studies were identified through citation tracking after the original search, indicating our search strategy was comprehensive. Unlike previous reviews, inclusion criteria for the review supported the examination of findings from studies conducted across a variety of clinical settings including specialty units or wards and ambulatory care. This helped in characterizing the use of ML-based prediction models in different patient-care environments with varying clinical endpoints. Wherever the original studies provided the data, comparisons were drawn between the performance of the ML models and that of aggregate-weighted EWS. This gives an indication of the differences in accuracy of the models in predicting clinical deterioration.

### Limitations

The findings within this review are subject to some limitations. First, the literature search, assessment of eligibility of full-text articles, inclusion in the review, and extraction of study data were carried out by only 1 author. Second, only the findings from published studies were included in this scoping review, which may affect the results due to publication bias. While studies from a variety of settings were included, the generalizability of our findings may be limited due to the heterogeneity of patient populations, clinical practices, and study methodologies. Sampling procedures and frequencies varied across studies from single to multiple observations of patient vital signs, and clinical outcome definitions were based on different criteria or aggregate-weighted EWS. Finally, due to this variation in ML methods, prediction windows, and outcome reporting, a meta-analysis was not feasible.

### Conclusion

Our findings suggest that ML-based EWS models incorporating easily accessible vital sign measurements are effective in predicting physiological deterioration in patients. Improved prediction performance was also observed with these models when compared to traditional aggregate-based risk stratification tools. The clinical impact of these ML-based EWS could be significant for clinical staff and patients due to decreased false alerts and increased early detection of warning signs for timely intervention, though further development of these models is needed and the necessary prospective research to establish actual clinical utility does not yet exist.

## Appendix

Multimedia Appendix 1 Search terms.

Multimedia Appendix 2 Description of ML methods and relevant terms.

Multimedia Appendix 3 Comparison between performance of ML based EWS and aggregate EWS.

## Abbreviations

AUROC: area under the receiver operating characteristic
AVPU: alert, verbal, pain, unresponsive
BP: blood pressure
ED: emergency department
EWS: early warning system
HR: heart rate
ICU: intensive care unit
MEWS: Modified Early Warning Score
MIMIC: Medical Information Mart for Intensive Care
ML: machine learning
NEWS: National Early Warning Score
PRISMA: Preferred Reporting Items for Systematic Reviews and Meta-Analyses
qSOFA: quick Sequential Organ Failure Assessment
RR: respiratory rate
TRIPOD: Transparent Reporting of a Multivariable Prediction Model for Individual Prognosis or Diagnosis
