# Supplementary material for: Machine Learning–Based Early Warning Systems for Clinical Deterioration: Systematic Scoping Review
Source: J Med Internet Res. 2021 Feb 4;23(2):e25187. doi: 10.2196/25187 (PMC7892287; doi:10.2196/25187)
Supplement: Multimedia Appendix 1 [file jmir_v23i2e25187_app1.docx]

## Appendix 1

### Search terms

(vital [All Fields] AND signs [All Fields]) AND

(clinical [All Fields]) OR (acute [All Fields]) OR (hospital [All Fields] OR (ambulatory [All Fields]) AND

(machine [All Fields] AND learning [All Fields]) OR (artificial [All Fields] AND intelligence [All Fields]) OR (deep [All Fields] AND learning [All Fields]) OR (neural [All Fields] AND network [All Fields]) OR (AI [All Fields]) OR (ML [All Fields]) AND

(deterioration [All Fields]) OR (early [All Fields] AND warning [All fields] OR early [All Fields] AND detection [All Fields] OR early [All Fields] AND diagnosis [All Fields] OR risk [All Fields] AND prediction [All Fields])
