# Supplementary material for: Machine Learning–Based Early Warning Systems for Clinical Deterioration: Systematic Scoping Review
Source: J Med Internet Res. 2021 Feb 4;23(2):e25187. doi: 10.2196/25187 (PMC7892287; doi:10.2196/25187)
Supplement: Multimedia Appendix 2 [file jmir_v23i2e25187_app2.docx]

## Appendix 2

### Description of ML methods and relevant terms

Machine learning is a sub-domain of artificial intelligence, which uses algorithms to train mathematical models using sample data in order to make predictions or decisions (most often a classification or regression) based on new data. Most machine learning approaches are either supervised, where an algorithm learns from a training data set that includes ‘true’ outputs (labelled data), or unsupervised, where an algorithm attempts to discover outputs on its own. This section provides a brief overview of the machine learning techniques (all supervised) that have been used for early warning systems.

### Naïve Bayes

Naïve Bayes classifiers are simple probabilistic classifiers based upon Bayes’ theorem that make the “naive” assumption of independence between explanatory variables [59]. A Naive Bayes classifier calculates the posterior probability of each possible class as the product of the prior probability and the likelihood function. The classifier then selects the class with the highest posterior probability as the predicted class.

The independence assumption in Naïve Bayes classifiers greatly simplifies the parameter estimation and contributes to the popularity of the algorithm. Naïve Bayes classifiers have also been shown to perform well in the presence of noise and irrelevant explanatory variables [59].

### Decision Tree

Decision tree classifiers create a series of decision rules based on explanatory variables to predict a categorical response variable [60]. To derive a decision tree, the algorithm applies a splitting rule on successively smaller partitions of data, with each partition being a node on the tree. The partition consisting of all data is the root node. Splits are selected to minimize some measure of node impurity or heterogeneity in each resulting partition. The splitting process repeats on each branch of the tree until additional splits yield no further reductions in node impurity, or some other prespecified stopping criterion is reached.

Decision trees are easy to understand, and they are also flexible to capture nonlinear effects and incorporate higher-order interactions between explanatory variables [60]. The most important limitations are that decision trees are highly sensitive to small perturbations in data and are prone to overfitting.

### Ensemble Methods (Random Forest, Adaptive Boosting and Gradient Boosting)

Ensemble methods, including bagging and boosting, combine the predictions of several base models to improve predictive performance in comparison with a single model. Bagging independently fits a base model to each bootstrap sample of the original data, and then creates a final prediction by averaging the predictions from all base models. It improves predictive performance by reducing model variance without affecting bias [61]. Random forest is an example of bagging which uses decision tree as base model. In order to further reduce model variance, the random forest algorithm only considers a random subset of explanatory variables when splitting each node in a decision tree [62].

By contrast, boosting fits base models in a sequential fashion and improves predictive performance by reducing model bias. In adaptive boosting (AdaBoost), initially, all samples are equally weighted, and the weights will be updated after each iteration; to constantly reduce prediction error, incorrectly classified samples in an iteration will receive increased weights, hence, subsequent iterations will prioritize correctly classifying these samples [63]. Gradient boosting is a generalization of AdaBoost that uses gradient descent to optimize any differentiable loss function [64].

### Kernel-based methods

In machine learning, a kernel is a similarity function between pairs of data-points. Kernel-based methods use the kernel trick to transform linear problems into nonlinear ones solely through the choice of kernel [65].

The most common kernel-based method is the support vector machine, an algorithm primarily used for classification [66]. In this setting, a kernel is defined between the hand-selected predictors. Common choices include the Gaussian (or squared exponential) kernel for continuous variables, while numerous kernels for categorical data have been proposed in the literature [67]; these kernels can be additively or multiplicatively combined under certain rules, allowing the model to flexibly consider predictors of mixed continuous and categorical types. SVMs minimize the hinge loss, which expresses the existence of a margin around the classification boundary.

### Neural networks

Artificial neural networks (ANNs) are mathematical models whose structures are inspired by the workings of the brain. Early work showed that they were incapable of efficiently approximating simple functions. However, recent advances in hardware and computational techniques have seen artificial neural networks, particularly deep neural networks, come to dominate certain tasks in machine learning. Deep learning generally refers to models called deep artificial neural networks (ANNs), and the family of gradient descent-based optimization algorithms used to train them [68]. ANNs consist of a sequence of layers, typically alternating layers which consist of a nonlinear function of a linear map. “Deep” is used to describe ANNs with a large number of layers. Deep neural networks are favoured in part because of their ability to automate the process known as feature engineering, which refers to the transformation of raw data into features which are useful for an algorithm to learn from.

### Model performance in ML

All included studies have used a number of different ML models for the classification task at hand. While all ML models have advantages and disadvantages in different scenarios, further research is required to establish which approach is best for predicting clinical deterioration. Model error can be attributed to ‘bias’ due to the model’s limitations in capturing different types of relationships; and ‘variance’ due to the noise in training data. While a high degree of bias could lead to the model underfitting, a high degree of variance could lead to overfitting. Therefore, it is key for ML models in this research area to strike a balance between bias, variance and clinical utility specific to the problem and setting.
