# Supplementary material for: Machine Learning–Based Early Warning Systems for Clinical Deterioration: Systematic Scoping Review
Source: J Med Internet Res. 2021 Feb 4;23(2):e25187. doi: 10.2196/25187 (PMC7892287; doi:10.2196/25187)
Supplement: Multimedia Appendix 3 [file jmir_v23i2e25187_app3.docx]

## Appendix 3: Comparison between performance of ML based EWS and aggregate EWS

| **Study** | **ML** | **NEWS** | **MEWS** | **SEDS** | **qSOFA** | **SOFA** | **SIRS** | **SAPS II** | **KTAS** | **TIMI** | **Hospital alarm system** |
| --- | --- | --- | --- | --- | --- | --- | --- | --- | --- | --- | --- |
| Badriyah et al., 2014  [45]  (Decision tree analysis) | Cardiac arrest AUROC: 0.708  Unanticipated ICU admission AUROC: 0.862  Death AUROC: 0.899  Any outcomes AUROC: 0.877 | Cardiac arrest AUROC: ﻿0.722  Unanticipated ICU admission AUROC: ﻿0.857  Death AUROC: ﻿0.894  Any outcomes AUROC: ﻿0.873 |  |  |  |  |  |  |  |  |  |
| Chiew et al., 2019  [23]  (Gradient boosting)  (30-day sepsis related mortality) | F1: 0.50  AUPRC: 0.35 | F1: 0.38,  AUPRC: 0.28 | F1: 0.30,  AUPRC: 0.25 | F1: 0.40, AUPRC: 0.22 | F1: 0.32,  AUPRC: 0.21 |  |  |  |  |  |  |
| Chiu et al., 2019  [42]  (Logistic regression)  (death, cardiac arrest, unplanned ICU admission) | 24h before event AUROC: 0.779  12h before event AUROC: 0.815  6h before event AUROC: 0.841 | 24h before event  AUROC: 0.754  12h before event AUROC: 0.789  6h before event AUROC: 0.813 |  |  |  |  |  |  |  |  |  |
| Desautels et al., 2016  [37]  (Insight classifier) | Sepsis at onset  AUROC: 0.880  APR: 0.6  Sepsis 4 hours before onset  AUROC: 0.74  APR: 0.28 |  | AUROC: 0.803, APR: 0.327 |  | AUROC: 0.772 APR: 0.277 | AUROC: 0.725  APR: 0.284 | AUROC: 0.609 APR: 0.160 | AUROC: 0.700 APR: 0.225 |  |  |  |
| Jang et al., 2019  [35]  (cardiac arrest) | ANN MLP AUROC: 0.929  LSTM AUROC: 0.933  Hybrid ANN AUROC: 0.936  Random forest AUROC: 0.923  Logistic regression AUROC: 0.914 |  | AUROC: 0.886 |  |  |  |  |  |  |  |  |
| Kwon et al., 2018  [26]  (cardiac arrest, death) | Recurrent neural networks AUROC: 0.85  AUPRC: 0.044.  Random forest  AUROC: 0.78  AUPRC: 0.014 |  | AUROC: 0.603  AUPRC: 0.003 |  |  |  |  |  |  |  |  |
| Kwon et al., 2018  [11]  (Deep learning model using multilayer perceptron)  (death, critical care admission, hospitalization) | AUROC: 0.935 AUPRC: 0.264 |  | AUROC: 0.810, AUPRC: 0.116 |  |  |  |  |  | AUROC: 0.785, AUPRC: 0.192 |  |  |
| Liu et al., 2014  [36]  (Ensemble learning model)  (cardiac arrest & death within 72h) | AUC: 0.812 |  | AUC: 0.622 |  |  |  |  |  |  | AUC: 0.637 |  |
| Mao et al., 2018  [34]  (Gradient tree boosting) | Sepsis onset AUROC: 0.92  Severe sepsis onset  AUROC: 0.87  Septic shock 4 hours before AUROC: 0.96  Severe sepsis 4 hours before AUROC: 0.85 | AUROC: 0.76 |  |  |  | AUROC: 0.65 | AUROC: 0.72 |  |  |  |  |
| Olsen et al., 2018  [46]  (random forest) | Accuracy: 92.2%; Sensitivity: 90.6%; Specificity: 93.0%;  AUROC: 96.9% |  |  |  |  |  |  |  |  |  | Number of false alarms decreased by 85%,  Number of missed Early Signs of Deterioration decreased by 73% compared to existing alarm system |
